# Protective and risk activities for emotional and behavioural well‐being of children and adolescents during the COVID‐19 lockdown

**DOI:** 10.1111/cch.13003

**Published:** 2022-03-21

**Authors:** Ilaria Nicolì, Maria Spinelli, Francesca Lionetti, Maria Grazia Logrieco, Mirco Fasolo

**Affiliations:** ^1^ Department of Neuroscience, Imaging and Clinical Sciences University G. D'Annunzio, Chieti‐Pescara Chieti

**Keywords:** activities, adolescents, children, COVID‐19, emotional and behavioural well‐being.

## Abstract

**Background:**

The lockdown imposed to contain the COVID‐19 pandemic brought deep changes in the daily life of Italian children and adolescents, increasing the time spent at home. This study aims to explore how activities that children and adolescents carried out at home during the lockdown were related to their emotional and behavioural well‐being.

**Method:**

Parents completed an anonymous online survey on how much time their children and adolescents dedicated to social networks, solitary screen time, play time and to a series of parent–child dyadic activities. They also reported on their offspring's emotional difficulties and behavioural problems.

**Results:**

The use of social networks had a negative impact on children's emotional difficulties, while it promoted well‐being in adolescents. Pertaining to solitary screen activities, these were associated with more behavioural problems in both children and adolescents. Regarding parent–child dyadic activities, get along with the parent was associated with less emotional difficulties and behavioural problems in children and with less behavioural problems in adolescents. Furthermore, for adolescents, the more they talked with the parent, the less behavioural problems they evidenced.

**Conclusion:**

The current study may help to identify activities that could be promoted and those that should be limited to effectively menage home time, in order to ultimately safeguard the emotional and behavioural well‐being of children and adolescents.

**Key messages**

- Social networks appeared to play a protective role for adolescents emotional well‐being, during the COVID‐19 lockdown.
- Social networks represent a risky activity for children, with an increase of emotional difficulties.
- Children and adolescents engaged in solitary screen time were more vulnerable to behavioural problems.
- Parents should limit solitary screen time in children and adolescents.
- Parents should encourage the sharing of quality time with children and adolescents.

## 1 INTRODUCTION

The lockdown imposed to contain the COVID‐19 pandemic brought deep changes in the lives of children and adolescents, as the closure of schools and educational facilities and an increase in time spent at home. Activities conducted at home were more limited and mostly carried out alone, or with the parent. These substantial changes in daily routines and activities had an impact on parents, and this, in turn, influenced children's well‐being and adjustment (Lionetti et al., 2022; Spinelli et al., 2020). The main aim of the present study was to investigate how and to what extend the activities performed at home during the lockdown by children and adolescents were related to their emotional and behavioural well‐being. Specifically, we focused on relevant at‐home activities such as time spent on social networks, solitary screen time, play time and on a set of different parent–child dyadic activities. Available research evidences reported that with the increase of time spent at home time, technology became essential to allow children and adolescents to interact with each other (Wiederhold, 2020). Indeed, social networks proved to be an ideal platform for children and adolescents to keep connection with peers during social distancing restrictions (Nagata et al., 2020). On the other hand, other studies provided evidence that time on social networks represents potentially a risky activity, especially for adolescent's self‐regulation, bringing psychosocial outcomes such as depression or anxiety (OKeeffe & Clarke‐Pearson, 2011).

Solitary screen‐time activities, including watching TV or playing videogames, also continued to rise among children and adolescents during the lockdown (Pietrobelli et al., 2020), with potential risks for emotion regulation competences in children (Domingues‐Montanari, 2017) and behavioural difficulties in adolescents (Twenge & Campbell, 2018). Besides the technological activities, during the pandemic restrictions children engaged in different play‐based activities, such as play with LEGO or play with siblings, with positive effect on their emotional well‐being (Graber et al., 2020). Indeed, a recent study indicated that play time was linked with positive emotions in children (Mondragon et al., 2021). Finally, with the highly increment of home time, parents suddenly became the children's and adolescents' key reference point (Spinelli et al., 2020) and were more involved in dyadic activities, such as playing and reading together (Lee et al., 2020). These sharing moments may play a protective factor for emotional and behavioural well‐being of children and adolescents (Pallini et al., 2018, 2019) during such a challenging period. Recent studies provided evidences that parent–child emotional closeness indeed promoted an overall positive adjustment to the pandemic, particularly in children potentially more vulnerable due to specific inherited, temperamental traits (Lionetti et al., 2022).

### 1 The current study

In sum, different studies highlighted how different types of home‐based activities differently impacted on children's and adolescents' well‐being. However, to the best of our knowledge, no study thus far has specifically examined what type of home‐based activities contributed, and in which direction, to the adjustment during the lockdown. Furthermore, no study has investigated the effect of this specific set of activities considering both children and adolescents. The purpose of this report is to explore separately in children and adolescents which activities affected their emotional and behavioural well‐being most, considering time on social networks, solitary screen time, play time, and different parent–child dyadic activities.

## 2 METHOD

### 2.1 Participants

The survey population was composed by 646 Italian children and their parents. Based on child age, participants were divided in two groups: 483 aged 5–10 years (*M* = 7.27, *SD* = 1.65; *F* = 53%), to represent the group defined as children, and 163 aged 11–17 years (*M* = 12.44, *SD* = 1.41; *F* = 46%), to represent the group defined of adolescents.

### 2.2 Procedure

Parents replied to an anonymous online survey, after having signed the written consent form and explicitly agreed to take part to the study. The survey was shared via social media 1 month after the beginning of the Covid‐19 first wave lockdown in Italy.

### 2.3 Study measures

#### 2.3.1 Activities performed by children and adolescents

Parents rated on an 8‐point scale (from 1 = *less than half an hour* to 8 = *seven hours or more*) how much time children and adolescents dedicated to a series of seven activities. Activities were grouped as follows: time on social networks (one item), referring to time spent on social media including WhatsApp, Facebook or Instagram; solitary screen time (three items) that included watching TV, playing with videogames and playing with the mobile phone; and play time (three items) that included playing alone, playing with a partner and reading a book with the parent.

### 2.4 Parent and child dyadic activities

Parents rated on a 5‐point scale (from 1 = *less than a day* to 5 = *every day*) how much time they spent with their children or adolescents on the following six activities: ‘Having fun together’, ‘Reading with him/her’, ‘Listening to him/her ideas’, ‘Getting along’, ‘Talking about the next day's activities’ and ‘Asking how he/she feels’.

#### 2.4.1 Children's and adolescent's emotional and behavioural well‐being

Parents completed the Italian version of the Strengths and Difficulties Questionnaire (SDQ; Goodman, 2001). In the current study, we included the subscales emotional difficulties and behavioural problems.

### 2.5 Plan of analysis

We computed four multivariate general linear models. We considered as dependent variables the SDQ emotional difficulties and behavioural problems scales, and as predictors the activities performed by children and adolescents, and the parent–child dyadic activities. The two categories of predictors were investigated separately in children and adolescents.

## 3 RESULTS

As reported in Table 1, in both groups of children and adolescents emerged a significant effect of time on social networks on SDQ emotional difficulties and of solitary screen time on SDQ behavioural problems. Particularly, in the children group, more time spent on social networks was associated with more emotional difficulties. Conversely, for the adolescent group, more time spent on social networks was associated with less emotional difficulties. In both groups, more solitary screen time activity was associated with more behavioural problems.

**TABLE 1:** Multivariate general linear model of activities performed by children and adolescents associated with strengths and difficulties questionnaire (SDQ) emotional and behavioural problems subscales scores

		B	Std. error	*t*	Sign.	Confidence interval 95%	
						Lower limit	Upper limit
Children – SDQ emotional difficulties	Time on social networks	0.50	0.16	3.01	.03	0.17	0.83
	Solitary screen time	0.04	0.08	0.55	.58	−0.11	0.20
	Play time	−0.04	0.04	−0.93	.35	−0.14	0.05
Children – SDQ behavioural problems	Time on social networks	−0.01	0.14	−0.10	.91	−0.31	0.27
	Solitary screen time	0.16	0.07	2.32	.02	0.02	0.30
	Play time	−0.04	0.04	−1.08	.28	−0.13	0.03
Adolescents – SDQ emotional difficulties	Time on social networks	−0.39	0.19	−2.09	.04	−0.78	−0.01
	Solitary screen time	0.14	0.10	1.32	.19	−0.07	0.36
	Play time	−0.05	0.09	−0.53	.59	−0.23	0.13
Adolescents – SDQ behavioural problems	Time on social networks	−0.25	0.16	−1.58	.11	−0.58	0.06
	Solitary screen time	0.25	0.09	2.75	.08	0.06	0.43
	Play time	−0.08	0.07	−1.05	.29	−0.24	0.07

Regarding parent–child dyadic activities, as described in Table 2, in both groups a significant effect of the activity ‘Getting along’ emerged on SDQ emotional difficulties and behavioural problems. Specifically, in the children group, the more they spent time ‘Getting along’ with their parent the less emotional difficulties and behavioural problems were reported. Similarly, in the group of adolescents, the more they spent time ‘Getting along’ with their parents the fewer behavioural problems were reported. Furthermore, in the group of adolescents, a significant effect of the activity ‘Talking with him/her about next day's activities’ emerged. Specifically, the more adolescent and parent ‘Talk about the next day's activities’, the fewer behavioural problems were reported.

**TABLE 2:** Multivariate general linear model of parent and child/adolescent dyadic activities associated with strengths and difficulties questionnaire (SDQ) emotional and behavioural problems subscales scores

		B	Std. error	*t*	Sign.	Confidence interval 95%	
						Lower limit	Lower limit
Children – SDQ emotional difficulties	Having fun together	−0.10	0.11	−0.91	.36	−0.32	0.12
	Reading with him/her	−0.00	0.07	−0.00	.99	−0.14	0.14
	Listening to him/her ideas	0.07	0.16	0.45	.65	−0.24	0.39
	Getting along	−0.46	0.11	−4.10	.01	−0.69	−0.24
	Talking with him/her about next days activities	−0.10	0.07	−1.42	.15	−0.25	0.04
	Asking how he/she feels	−0.01	0.08	−0.16	.86	−0.18	0.15
Children – SDQ behavioural problems	Having fun together	−0.11	0.09	−1.20	.23	−0.29	0.07
	Reading with him/her	0.00	0.06	0.03	.97	−0.11	0.12
	Listening to him/her ideas	−0.08	0.13	−0.61	.53	−0.35	0.18
	Getting along	−0.64	0.09	−6.84	.00	−0.82	−0.45
	Talking with him/her about next days activities	−0.05	0.06	−0.90	.36	−0.17	0.06
	Asking how he/she feels	−0.03	0.06	−0.52	.60	−0.17	0.10
Adolescents – SDQ emotional difficulties	Having fun together	−0.12	0.14	−0.84	.40	−0.41	0.16
	Reading with him/her	0.26	0.14	1.86	.06	−0.01	0.53
	Listening to him/her ideas	−0.46	0.24	−1.86	.06	−0.95	0.02
	Getting along	−0.26	0.20	−1.28	.20	−0.67	0.14
	Talking with him/her about next days activities	−0.24	0.15	−1.56	.12	−0.54	0.06
	Asking how he/she feels	−0.13	0.14	−0.97	.33	−0.41	0.14
Adolescents – SDQ behavioural problems	Having fun together	−0.04	0.11	−0.43	.66	−0.27	0.17
	Reading with him/her	0.01	0.10	0.14	.88	−0.19	0.22
	Listening to him/her ideas	−0.00	0.19	−0.02	.98	−0.38	0.37
	Getting along	−0.58	0.15	−3.66	.01	−0.89	−0.26
	Talking with him/her about next days activities	−0.33	0.11	−2.82	.01	−0.57	−0.10
	Asking how he/she feels	−0.04	0.10	−0.40	.68	−0.2	0.17

## 4 DISCUSSION

The current study provides a snapshot of how activities performed by children and adolescents during the lockdown were related to their emotional and behavioural well‐being. Three major themes emerged. First, in our study social networks appeared to play a protective role for emotional well‐being, but only for adolescents. This is an unexpected and revealing result. Probably, with school closures and in the absence of face‐to‐face interaction with peers and friends, adolescents welcomed the advantages that technology offers for social connectivity (Goldschmidt, 2020). On the contrary, time spent on social networks were found to represent a risky activity for children's emotional well‐being, with an increase of emotional difficulties. A possible explanation is that an increase on time spent on social network in children may have reduced the time spent on those positive parent–child interactions (Bozzola et al., ) that constitute, for younger children, the elective context to learn and consolidate emotion regulation competences (Tronik et al., 1998).

Second, in line with prior research (Domingues‐Montanari, 2017; Stiglic & Viner, 2019), we found that children and adolescents engaged in solitary screen time activities were more vulnerable to behavioural problems. Hence, there are reasons to be concerned about the impact of screen time for its deleterious effects on irritability and low mood (Domingues‐Montanari, 2017; Lissak, 2018).

Third, we found that the sharing of activities between parents and children played a protective effect on emotional and behavioural well‐being. Specifically, our results indicated that a parent–child relationship characterized by harmony and reconciliation was able to promote children's emotional and behavioural well‐being. Getting along with the parent, establishing frequent and positive verbal exchanges in a context of understanding and support was protective for the adjustment to the pandemic (see also Gambin et al., 2020). This is in line with theoretical reasoning and evidences proving that, for adolescents, a relationship with the parent characterized by open communication is able to foster a sense of understanding with important consequences for well‐being (Kobak et al., 2017).

## 5 CONCLUSION AND FUTURE DIRECTIONS

Before concluding, it is important to acknowledge that the current study is not without limitations. The main limitation concerns the way in which the data were collected, that is, through parents‐report questionnaire. However, this was the only applicable method for data collection when the first COVID‐19 lockdown was issued. Besides, we did not explore the full range of possible child and adolescent activities. Future research should include the point of view of children and adolescents as well, to deepen our understanding of this topic and expanding the range of activities to be considered in such type of studies. Notwithstanding these limitations, we believe that our study points out for the possibility to prevent the onset of emotional and behavioural problems by promoting specific home‐based activities that, even during extreme contexts as the COVID‐19 lockdown, are able to promote well‐being of children and adolescents. This is even more relevant if we consider that many studies have demonstrated the stability of behavioural problems over time (e.g., Robins, 1991) and a strong association between behavioural problems and later psychopathology (e.g., Caspi et al., 1996; Robins, 1991). To conclude, considering the deep changes that the lockdown and COVID‐19 brought to the daily life of children and adolescents, with an increase in time spent at home even during transition periods when restrictions were partially released, we believe that this study could give an important contribution to the identification of helpful and protective activities to be promoted to support positive emotion regulation competences and decrease the risk of behavioural problems during potentially challenging periods.

## CONFLICT OF INTEREST

No potential competing interest was reported by the authors.

## ETHICS STATEMENT

The study was approved by the ethical commitment of the Department and was conducted according to American Psychological Association guidelines in accordance with the 1964 Helsinki Declaration.

## Data Availability

The data that support the findings of this study are available from the corresponding author upon reasonable request.

## References

[cch13003-bib-0001] Bozzola, E. , Spina, G. , Ruggiero, M. , Memo, L. , Agostiniani, R. , Bozzola, M. , & Villani, A. (2018). Media devices in pre‐school children: The recommendations of the Italian pediatric society. Italian Journal of Pediatrics, 44(1), 1–5. 29898749 10.1186/s13052-018-0508-7 PMC6001217

[cch13003-bib-0025] Caspi, A. , Moffitt, T. E. , Newman, D. L. , & Silva, P. A. (1996). Behavioral observations at age 3 years predict adult psychiatric disorders longitudinal evidence from a birth cohort. Archives of General Psychiatry, 53(11), 1033–1039. https://jamanetwork.com/journals/jamapsychiatry/article-abstract/497677 8911226 10.1001/archpsyc.1996.01830110071009

[cch13003-bib-0002] Domingues‐Montanari, S. (2017). Clinical and psychological effects of excessive screen time on children. Journal of Paediatrics and Child Health, 53(4), 333–338. 10.1111/jpc.13462 28168778

[cch13003-bib-0003] Gambin, M. , Woźniak‐Prus, M. , Sekowski, M. , Cudo, A. , Pisula, E. , Kiepura, E. , Boruszak‐Kiziukiewicz J. , & Kmita, G. (2020). Factors related to positive experiences in parent‐child relationship during the COVID‐19 lockdown. The role of empathy, emotion regulation, parenting self‐efficacy and social support. 10.1111/famp.12856 36724769

[cch13003-bib-0004] Goldschmidt, K. (2020). The COVID‐19 pandemic: Technology use to support the wellbeing of children. Journal of Pediatrics Nursing, 53, 88–90. 10.1016/j.pedn.2020.04.013 PMC7161478 32317129

[cch13003-bib-0005] Goodman, R. (2001). Psychometric properties of the strengths and difficulties questionnaire. Journal of the American Academy of Child & Adolescent Psychiatry, 40(11), 1337–1345. 10.1097/00004583-200111000-00015 11699809

[cch13003-bib-0006] Graber, K. M. , Byrne, E. M. , Goodacre, E. J. , Kirby, N. , Kulkarni, K. , O'Farrelly, C. & Ramchandani, P. G. (2020). A rapid review of the impact of quarantine and restricted environments on children's play and the role of play in children's health. Child: care, health and development. 10.1111/cch.12832 PMC7753247 33238034

[cch13003-bib-0007] Kobak, R. , Abbott, C. , Zisk, A. , & Bounoua, N. (2017). Adapting to the changing needs of adolescents: Parenting practices and challenges to sensitive attunement. Current Opinion in Psychology, 15, 137–142. 10.1016/j.copsyc.2017.02.018 28813254 PMC5886742

[cch13003-bib-0008] Lee, S. J. , Ward, K. P. , Chang, O. D. , & Downing, K. M. (2020). Parenting activities and the transition to home‐based education during the COVID‐19 pandemic. Children and Youth Services Review, 122(July 2020), 105585. 33071407 10.1016/j.childyouth.2020.105585 PMC7553006

[cch13003-bib-0009] Lionetti, F. , Spinelli, M. , Moscardino, U. , Ponzetti, S. , Garito, M. C. , Dellagiulia, A. , Aureli, T. , Fasolo, M. , & Pluess, M. (2022). The interplay between parenting and environmental sensitivity in the prediction of childrens externalizing and internalizing behaviors during COVIID‐19. Development and Psychopathology, 8(Mar 2022), 1–14. 10.1017/S0954579421001309 35256026

[cch13003-bib-0010] Lissak, G. (2018). Adverse physiological and psychological effects of screen time on children and adolescents: Literature review and case study. Environmental research, 164, 149–157. 29499467 10.1016/j.envres.2018.01.015

[cch13003-bib-0011] Mondragon, N. , Berasategi Sancho, N. , Dosil Santamaria, M. , & Eiguren Munitis, A. (2021). Struggling to breathe: A qualitative study of childrens wellbeing during lockdown in Spain. Psychology & Health, 36(2), 179–194. 10.1080/08870446.2020.1804570 32762551

[cch13003-bib-0012] Nagata, J. M. , Abdel Magid, H. S. , & Pettee Gabriel, K. (2020). Screen time for children and adolescents during the coronavirus disease 2019 pandemic. Obesity, 28(9), 1582–1583. 10.1002/oby.22917 32463530 PMC7283714

[cch13003-bib-0013] OKeeffe, G. S. , & Clarke‐Pearson, K. (2011). Clinical report: The impact of social media on children, adolescents, and families. Pediatrics, 127(4), 800–804. 10.1542/peds.2011-0054 21444588

[cch13003-bib-0014] Pallini, S. , Chirumbolo, A. , Morelli, M. , Baiocco, R. , Laghi, F. , & Eisenberg, N. (2018). The relation of attachment security status to effortful self‐regulation: A meta‐analysis. Psychological Bulletin, 144, 501–531. 10.1037/bul0000134 29517260

[cch13003-bib-0015] Pallini, S. , Morelli, M. , Chirumbolo, A. , Baiocco, R. , Laghi, F. , & Eisenberg, N. (2019). Attachment and attention problems: A meta‐analysis. Clin. Psychol. Res., 74, 101772. 10.1016/j.cpr.2019.101772 31739122

[cch13003-bib-0016] Pietrobelli, A. , Pecoraro, L. , Ferruzzi, A. , Heo, M. , Faith, M. , Zoller, T. , Antoniazzi, F. , Piacentini, G. , Fearnbach, S. N. , & Heymsfield, S. B. (2020). Effects of covid‐19 lockdown on lifestyle behaviors in children with obesity living in Verona, Italy: A longitudinal study. Obesity, 28, 1382–1385. 10.1002/oby.22861 32352652 PMC7267384

[cch13003-bib-0024] Robins, L. N. (1991). Conduct Disorder. The Journal of Child Psychology and Psychiatry, 32(1), 193–212. 10.1111/j.1469-7610.1991.tb00008.x 2037645

[cch13003-bib-0018] Spinelli, M. , Lionetti, F. , Setti, A. , & Fasolo, M. (2020). Parenting stress during the COVID‐19 outbreak: Socioeconomic and environmental risk factors and implications for children emotion regulation. Family process, 60, 639–653. 10.1111/famp.12601 32985703

[cch13003-bib-0019] Stiglic, N. , & Viner, R. M. (2019). Effects of screen time on the health and well‐being of children and adolescents: A systematic review of reviews. BMJ Open, 9(1), e023191. 10.1136/bmjopen-2018-023191 PMC6326346 30606703

[cch13003-bib-0020] Tronick, E. Z. , Bruschweiler‐Stern, N. , Harrison, A. M. , Lyons‐Ruth, K. , Morgan, A. C. , Nahum, J. P. , Sander, L. , & Stern, D. N. (1998). Dyadically expanded states of consciousness and the process of therapeutic change. Infant Mental Health Journal: Official Publication of the World Association for Infant Mental Health, 19(3), 290–299. 10.1002/(SICI)1097-0355(199823)19:3<290::AID-IMHJ4>3.0.CO;2-Q

[cch13003-bib-0021] Twenge, J. M. , & Campbell, W. K. (2018). Associations between screen time and lower psychological well‐being among children and adolescents: Evidence from a population based study. Preventive Medicine Reports, 12, 271–283. 10.1016/j.pmedr.2018.10.003 30406005 PMC6214874

[cch13003-bib-0022] Wiederhold, B. K. (2020). Children's screen time during the COVID‐19 pandemic: Boundaries and etiquette. Cyberpsychology, Behavior, and Social Networking, 23(6), 359–360. 10.1089/cyber.2020.29185.bkw 32437623

